# Children’s Interpretation of Ambiguous *wh*-Adjuncts in Mandarin Chinese

**DOI:** 10.3389/fpsyg.2020.01781

**Published:** 2020-07-28

**Authors:** Jing Li, Peng Zhou

**Affiliations:** Department of Foreign Languages and Literatures, Child Cognition Lab, Tsinghua University, Beijing, China

**Keywords:** *Wh*-adjuncts, syntactic cues, semantic interpretations, Mandarin Chinese, child language

## Abstract

The paper reports two studies investigating children’s acquisition of the *wh*-adjunct *zenme* in Mandarin. Unlike other Mandarin *wh*-words that correspond to a single meaning, *zenme* can be used to question either the manner or the cause of an event. Study 1 explored whether children understand that *zenme* is ambiguous between a causal and a manner reading. Study 2 examined whether they can use syntactic cues to disambiguate the two readings. The findings show that children as young as 4 years of age access both the manner and the causal reading, but they prefer the former over the latter. Children exhibit a developmental trajectory when acquiring the mapping relations between the syntactic positions of *zenme* and its corresponding semantic interpretations: 5-year-olds can use syntactic cues to disambiguate the two readings; 3-year-olds, however, are still in the stage of working out how the syntactic positions are mapped onto the relevant semantic interpretations; the critical change occurs at around 4 years of age. The implications of the findings were then discussed in relation to the two major competing theories of child language acquisition.

## Introduction

There are two major competing approaches to child language acquisition: the UG (Universal Grammar)-based approach and the usage-based approach. The UG-based approach is based on the theory of Universal Grammar by Noam Chomsky (1965). This approach emphasizes the discrepancy between language input and the linguistic knowledge children acquire in the first few years of life. In other words, the approach acknowledged the fact that the linguistic knowledge acquired by young children vastly exceeds the linguistic input they have been exposed to. To account for the discrepancy, the UG-based approach proposes that children are born with some abstract linguistic knowledge that guides their language acquisition. These innately specified linguistic constraints constitute the initial state of language acquisition, and hence form the basis on which knowledge of language develops (Chomsky, 1975, 1980). On this approach, sentences are hierarchically structured, and the interpretations that can be assigned to sentences are dependent on the abstract structural constraints. Guided by these constraints, children are expected to acquire language in a relatively rapid and effortless manner (Chomsky, 1980; Crain and Nakayama, 1987; Crain and Pietroski, 2001; Crain et al., 2017).

The usage-based approach, in particular the one represented by the constructivist theory, proposes that our grammatical knowledge consists in an inventory of different constructions learned from the input. Each construction is associated with a particular function, and children learn different constructions alongside their functions in the context (Goldberg, 2003, 2006; Ambridge and Lieven, 2011). This approach denies that children are born with innate linguistic knowledge, and claims that children learn language by witnessing language in use in linguistic contexts. On this approach, children acquire linguistic knowledge by attending to linguistic input and by using domain-general learning mechanisms, such as imitation, analogy and distributional analysis (Bybee, 2001; Pullum and Scholz, 2002; Tomasello, 2006; Lieven and Tomasello, 2008; Saxton, 2010). This approach expects that children acquire language in a more gradual and piecemeal fashion and that the acquisition of particular linguistic constructions relies heavily on the specific language to which a particular child is exposed (Tomasello, 2000, 2003). So one basic assumption following the usage-based approach is that more frequent constructions in the language input are acquired earlier than less frequent ones (Tomasello, 2000, 2003, 2006; Lieven and Tomasello, 2008; Ambridge and Lieven, 2011).

This paper reports two experimental studies on young children’s acquisition of two types of *zenme* (roughly corresponding to English *how*) questions in Mandarin Chinese, with an attempt to show how theoretical analyses of linguistic structures could raise interesting questions for child language acquisition, and how data from child language acquisition can, in turn, inform linguistic theories.

## *Zenme* Questions in Mandarin

Mandarin *zenme*, roughly corresponding to English *how*, is a commonly used interrogative adverb. Unlike many other Mandarin *wh*-words that correspond to a single meaning (e.g., *shei* ‘who,’ *nali* ‘where’), *zenme* can be used to question either the manner or the cause of an event (Wang, 1943; Ding, 1961; Lü, 1980; Zhu, 1982; Peng, 1993; Shao, 1996; Tsai, 1999, 2000, 2007, 2008; Xiao, 2009). We refer to the two uses of *zenme* as manner *zenme* and causal *zenme*, respectively. For instance, when *zenme* occurs in serial verb constructions as in (1) or PP + VP constructions as in (2), it is ambiguous between a causal reading and a manner reading (Peng, 1993; Shao, 1996). Interestingly, the ambiguity between manner *zenme* and causal *zenme* disappears in certain syntactic structures. For example, when *zenme* is preceded by verbal modifiers such as the temporal adverbial *jingchang* ‘often’ [see (3)], it receives the manner reading, whereas when it precedes these verbal modifiers, it obtains the causal reading, as in (4) (Peng, 1993; Shao, 1996; Tsai, 1999, 2000, 2007, 2008).


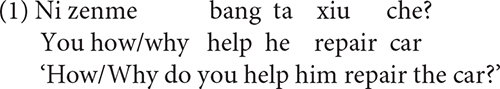



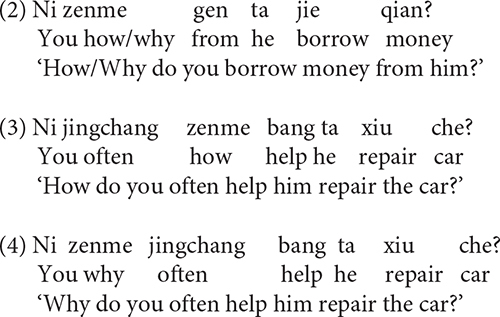


Causal *zenme* and manner *zenme* also exhibit asymmetries when they interact with modal verbs (Zhu, 1982; Peng, 1993; Shao, 1996; Tsai, 1999, 2000, 2007, 2008). Consider a typical modal verb *hui* ‘will/would,’ for example. *Hui* ‘will/would’ expresses future tense or possibility. When *zenme* is structurally lower than *hui*, it indicates a manner; when *zenme* is structurally higher than *hui*, it denotes a cause (Shao, 1996; Tsai, 1999, 2000, 2007, 2008). Therefore, (5) questions the manner or instrument, by which you help him repair the car; by contrast, (6) questions the cause or reason, for which you help him repair the car.


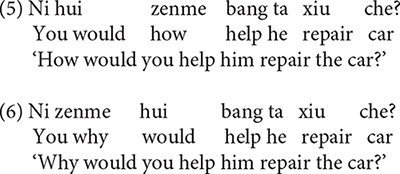


In the framework of the UG theory, Tsai (1999, 2000, 2007, 2008) attributed the distinct readings of *zenme* to their corresponding syntactic positions. Tsai’s analysis is based on the cartographic approach by Rizzi (1997, 1999, 2004) and Cinque (1999). According to Rizzi (1997), syntactic structures of clauses can be divided into three layers: the lexical layer, the inflectional layer and the complementizer layer. The lexical layer is headed by the verb and theta roles are assigned in this layer. The inflectional layer is headed by functional morphological specifications of the verb; case and agreement are licensed in this layer. The complementizer layer corresponds to the left periphery; topic, focus, interrogative and relative pronouns and some other functional categories are distributed in this layer, and thus this layer is often referred to as the split CP, as schematized in (7).

(7) Force Top^∗^ Int Top^∗^ Focus Mod^∗^ Top^∗^ Fin IP

Following this approach, Tsai (2008) argued that modal verbs relate to tense elements in the inflectional layer, and since causal *zenme* is hierarchically higher than modals, it is the sentential adverbial in the complementizer layer and merges directly into the left periphery. According to Tsai, causal *zenme* scopes over the entire IP and takes the corresponding event/state as its complement. The relatively lower manner *zenme* is a vP-modifier, which functions as the restrictive predicate of the underlying event argument associated with vP periphery, namely the lexical layer. Due to their syntactic positions, the interrogative scope of causal *zenme* is wider than modal verbs, and thus questions the causality of the event; whereas the interrogative scope of manner *zenme* is narrower than modals, so it questions the comitativity of the action, namely the manner and the way in which certain action is performed. The topography of Mandarin *zenme* adverbials is schematized in (8). More specifically, when *zenme* is structurally higher than the modal verb, it acts as the sentential outer adverbial, scoping over the entire IP and yielding a causal reading; when *zenme* locates between the modal verb and vP, it functions as a vP-modifier, giving rise to a manner reading.


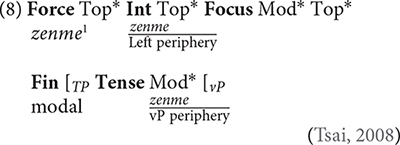
^1^

## *Wh*-Adjuncts in Child Language

Previous theoretical work on *zenme* provides detailed descriptions and insightful analyses of the syntactic distributions and the corresponding semantic interpretations of *zenme*. However, to the best of our knowledge, only a few studies investigated children’s acquisition of *zenme* in Mandarin. Prior research mainly used naturalistic data and reported that 4- and 5-year-old Mandarin-speaking children use *zenme* to ask both manner questions and causal questions (Li and Chen, 1997a, b; Kong and Chen, 1999). There is only one experimental study by Li et al. (2015) that directly examined Mandarin-speaking children’s knowledge of the two interpretations of *zenme*. They found that in both comprehension (i.e., a picture identification task) and production (i.e., a question task), 4- to 6-year-old children showed no significant difference in response to the manner questions containing the “*hui* + *zenme*” sequence as opposed to the causal questions containing the “*zenme* + *hui*” sequence, but children performed slightly better with the latter. On the basis of the findings, they argued that Mandarin-speaking children have more difficulties in acquiring the manner questions than the causal questions. However, we wish to point out a few potentially serious problems with their study concerning test materials, results and data interpretation. First, the test materials in the picture identification task were strongly ambiguous. The test pictures that were supposed to be designed to match the causal reading actually depicted two concurrent events corresponding to the causal and manner reading, respectively. Depicting two events in one picture might have strengthened the visual salience of these pictures as compared to those that only described one event in the manner reading condition. This visual salience might have significantly encouraged children to choose these pictures even when hearing the manner questions, because one of the events in these pictures corresponded to the manner reading anyway. This picture identification task, therefore, might have seriously underestimated children’s comprehension of the manner questions. Regarding the production task, the task design did not meet the felicity condition to elicit causal *zenme* questions, in particular the counter-expectation prerequisite, namely, it is only felicitous to ask a causal *zenme* question when an on-going event contradicts the speaker’s expectation, thereby leading the speaker to ask what has led to the unexpected event (Tsai, 2008). It has been well established that in order to test young children’s linguistic knowledge, the test contexts must meet the felicity conditions on the use of the target structure, which otherwise might seriously undermine children’s linguistic knowledge (Hamburger and Crain, 1982; Crain and Thornton, 1998; Gualmini, 2004, 2005; Gualmini et al., 2008). In addition, concerning the results and data interpretation, although there was slight difference in children’s proportions of correct answers in response to the two types of questions, the difference did not reach statistical significance in both the comprehension and production task, and thus has no statistical meaning.

To examine children’s acquisition of *wh*-adjuncts cross-linguistically, prior research found that English-speaking children seemed to exhibit asymmetrical patterns in the acquisition of *how* and *why* questions. It has been reported that English-speaking children acquired *why* questions later than *how* questions, and in fact among all *wh*-phrases *why* is the last to show full mastery of subject-auxiliary inversion (de Villiers, 1991; Rowland and Pine, 2000; Rowland et al., 2005; Thornton, 2008). The late acquisition of *why* questions led some researchers to propose that English-speaking children initially have different structural representations of questions from adults (de Villiers, 1991; Thornton, 2008). For instance, de Villiers (1991) proposed that English-speaking children do not initially project a CP phrase, and thus instead of moving a *wh*-phrase to [Spec, CP], which triggers subject-auxiliary inversion, they simply adjoin the *wh*-phrase to [Spec, IP], which does not trigger the inversion. According to de Villiers (1991), the linguistic input that triggers children to reanalyze the position of *wh*-phrases as in [Spec, CP] position is the embedded questions with a *wh*-phrase that has been moved from the original position to the intermediate [Spec, CP] position, as in the example *John wondered what Bill ate last night*. It has been argued that *why* lags behind *how* and other *wh-words* in children’s reanalysis process, which, according to de Villiers (1991), was presumably due to the fact that the triggering evidence of embedded *why* questions is limited in parental input. However, as de Villiers (1991) also pointed out, input alone is not sufficient to account for children’s errors of subject-auxiliary inversion, but rather the fundamental source of children’s non-adult *why* questions is their non-adult structural representations.

To take stock, it is interesting and important to study this *how/why* asymmetry from a cross-linguistic perspective in order to identify language universal and language-specific patterns underlying children’s acquisition of *wh*-adjuncts. However, on the basis of such limited prior research on Mandarin, it is difficult to draw any conclusive generalizations about Mandarin-speaking children’s acquisition of the two readings of *zenme*, in particular the contrast between causal *zenme* and manner *zenme* in complex structures containing modal verbs. To address these unresolved issues, the present paper reports two experimental studies that aimed to investigate Mandarin-speaking children’s understanding of the two readings of *zenme*, and in particular, we were interested to see whether young children can make use of the structural relation between *zenme* and modal verbs to distinguish between the two readings. As discussed, there are two major competing approaches to child language acquisition: the UG-based approach and the usage-based approach. What is crucial for the present paper is that the two approaches make different predictions about Mandarin-speaking children’s acquisition of the two readings of *zenme*, in particular in structures that involve hierarchical relations between *zenme* and the modal verb *hui*. Therefore, the present findings can, to some extent, inform us about the debate between the two approaches. The usage-based approach would predict that the acquisition of the two readings of *zenme* does not involve hierarchical structures. Rather, the two uses of *zenme* would be treated as separate constructions associated with separate functions, and the acquisition of the two uses of *zenme* depends heavily on the linguistic input children are being exposed to. Thus, on this account the more frequent use of *zenme* in the linguistic input should be acquired earlier than the less frequent one, and children do not rely on hierarchical structures to understand the two readings of *zenme*. By contrast, on the UG-based account children are expected to acquire the two uses of *zenme* by mastering the mapping relations between its syntactic positions and the corresponding semantic interpretations. More specifically, following Tsai’s (2008) analysis with the UG framework, the manner reading is related to VP in the lexical layer of the structure, whereas the causal reading involves CP, namely the left periphery, of the structure. Therefore, to correctly derive the causal and manner readings of *zenme*, children need, first, to get the syntactic positions of *zenme* right, and then to map the syntactic positions onto the corresponding semantic interpretations. According to the UG-based approach, children rely on hierarchical structures to acquire the two uses of *zenme* and they might exhibit asymmetries in the acquisition of the two uses of *zenme*, due to their non-adult structural representations of *zenme*. This prediction is based on previous research that observed an asymmetry in the acquisition of higher syntactic structures related to the CP layer versus the acquisition of lower syntactic structures relevant to the IP and VP layers. For example, Platzack (2001) reported that Swedish-speaking and German-speaking young children produced the syntax of lower structural levels (the IP and VP layers) in an adult-like manner, but they have problems producing the syntax of the higher structural level (the CP layer). Findings in support of children’s difficulty with the syntax of the CP layer at the left periphery have also been observed in Catalan-speaking, English-speaking, French-speaking, Greek-speaking and Spanish-speaking children (Rizzi, 1994; Müller et al., 1996; Radford, 1996; Grinstead, 1998; Marinis, 2004; Spinner and Grinstead, 2006). For instance, Spinner and Grinstead (2006) found that German-speaking children acquired constructions that involve the CP layer (i.e., fronted-object constructions and *wh*-questions) significantly later than those that do not involve the CP layer. Spinner and Grinstead (2006) dubbed this as the Left Peripheral Delay. According to this proposal, children acquire constructions that involve the CP layer of the left periphery relatively late, because the CP layer is proposed to be relevant to discourse functions, like topic/focus and interrogatives (Rizzi, 1997; Poletto, 2000; Benica’ and Poletto, 2004), and the integration of discourse/pragmatics functions into syntax/semantics takes time to develop. In other words, the mapping between syntax/semantics and discourse/pragmatics is not well established until at a relatively late stage of language development. Young children do not quite have an adult-like representation of the CP layer that is associated with discourse functions.

Note that in the current study the derivation of the two types of *zenme* questions involves different structural layers, the causal *zenme* question involves the CP layer and the manner *zenme* question relates to the VP layer. We were interested to find out whether Mandarin-speaking children also exhibited a similar asymmetry as observed in other languages. In the following sections, we first present two experimental studies that investigated Mandarin-speaking children’s understanding of the two types of *zenme* questions, and then we discuss which approach better explains the experimental findings.

## Experiment 1

Experiment 1 was designed to see whether Mandarin-speaking children interpret *zenme* ambiguously between a causal reading and a manner reading, and whether they exhibit a preference for one reading over the other. As discussed, both the causal reading and the manner reading are allowed when *zenme* occurs in serial verb constructions [see (1)] and PP + VP constructions [see (2)]. We were interested to find out how children interpret *zenme* in these two structures when both readings are made available in the context.

### Participants

Forty monolingual Mandarin-speaking children participated in Experiment 1: twenty 4-year-olds (11 boys and 9 girls, age range 4;0–4;11, mean 4;6), and twenty 5-year-olds (13 boys and 7 girls, age range 5;0–5;9, mean 5;4). The child participants were recruited from Beijing Taolifangyuan Kindergarten and had no reported history of speech, hearing or language disorders. In addition, 18 Mandarin-speaking adults (age range 18–31, mean 25) were tested as controls. They were students at Tsinghua University, and had no self-reported speech or hearing disorders.

### Materials and Design

We used a Question–Answer task, an extension of the Truth Value Judgment Task by Crain and Thornton (1998). Four test stories were constructed. For each story, one test sentence and one filler sentence were created. So there were four test sentences and four filler sentences in total. See Appendix A for all the test and filler sentences. Two of the test sentences used PP + VP constructions [see (9)] and two used serial verb constructions [see (10)]. As discussed, in both constructions *zenme* is ambiguous between a causal reading and a manner reading.


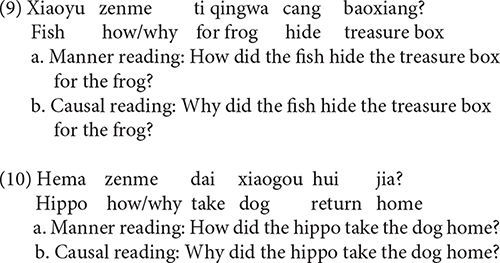


The test sentences were produced by a female native speaker of Beijing Mandarin. Note that prosodic cues (i.e., pitch accent) can be used to distinguish causal *zenme* from manner *zenme*. Specifically, when *zenme* is accented, the sentence expresses a manner question; by contrast, if other elements instead of *zenme* is accented, the sentence asks a causal question (Zhu, 1982; Peng, 1993; Shao, 1996). Thus, in order to control for potential prosodic effects on children’s interpretation of *zenme* questions, the speaker was asked to produce the test sentences using the same intonation pattern (i.e., level intonation) and with no words being accented. A post-recording survey was conducted to make sure that the test sentences were produced successfully. Eighteen Mandarin-speaking adults participated in this survey, where they were presented with each recorded sentence and were asked to judge whether the sentence has level intonation and if any particular word was accented in the sentence. The findings were that all the test sentences were judged to be in level intonation, and no words in any of the test sentences were judged to be accented.

A typical trial is used to illustrate the test scenario. On this trial, the experimenter acted out a story about a fish and a frog in Mandarin. The English translation of the story is given as follows.

**FIGURE 1:**
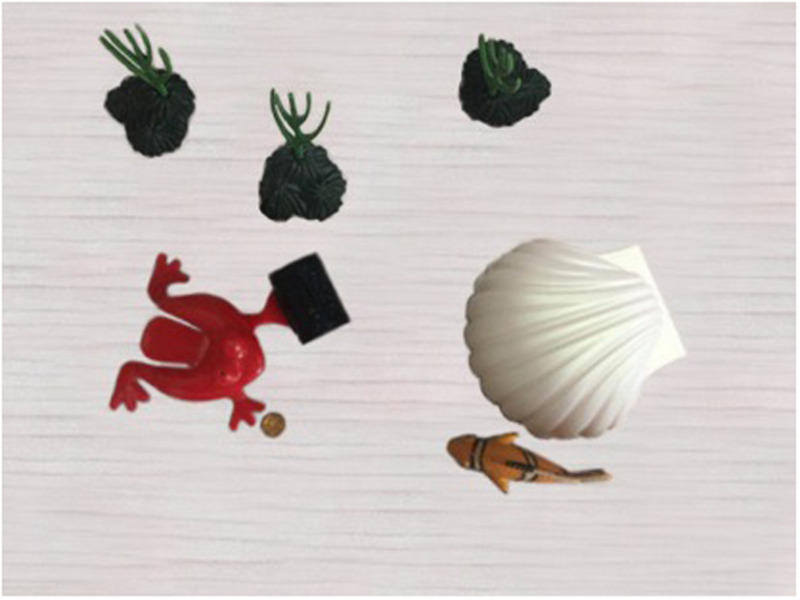
The story scene corresponding to the causal reading.

**FIGURE 2:**
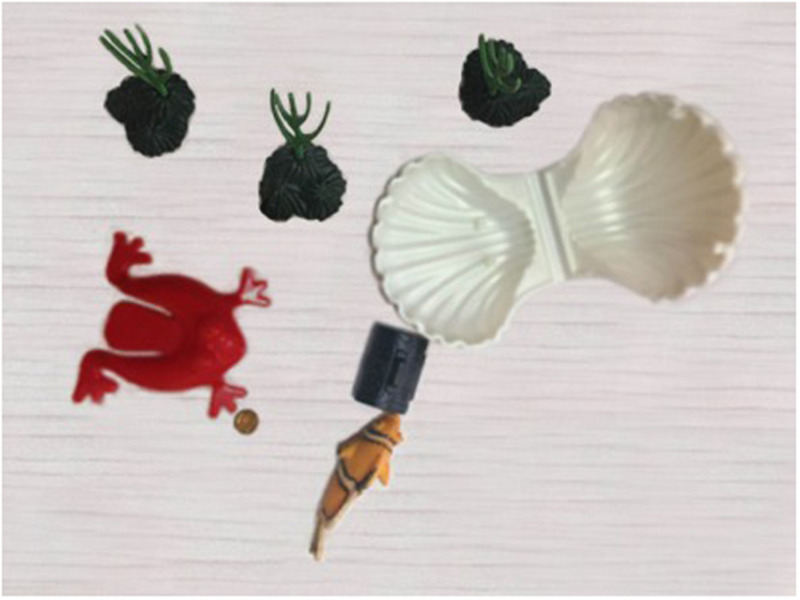
The story scene corresponding to the manner reading.

This is a story about a fish and a frog. They live in the same village, but the fish does not like the frog, because the frog always comes to bully the fish. The fish decides never to talk to the frog. One day the frog finds a treasure box and he wants to hide it. But the frog cannot lift the treasure box, because it is too heavy. At this time, the fish passes by. The frog walks to the fish and asks him to help hide the treasure box. At first, the fish does not want to help the frog, because he does not like the frog. Then the frog comes up with an idea. He knows that the fish likes gold coins. So he takes out a gold coin and says to the fish “Could you help me hide the treasure box? I can give you a gold coin.” The fish loves gold coins and cannot resist the temptation of getting a gold coin, so he changes his mind and decides to offer his help (**Note that the causal reading is fulfilled at the point, see Figure 1**). The frog and the fish lift the treasure box together and they start to look for a place to hide the treasure box. The frog asks the fish where they should hide it. The fish suggests that they hide it into his shell. The fish then opens his shell and puts the treasure box into it (**Note that the manner reading is established at this point, see Figure 2**). The frog gives the fish a gold coin as promised.

Figure 1 illustrates the scene corresponding to the causal reading of *zenme*. Figure 2 displays the scene corresponding to the manner reading of *zenme*.

We wish to highlight three design features. First, as discussed earlier, in order to test young children’s linguistic knowledge, the test contexts must meet the felicity conditions on the use of the target structure, which otherwise might seriously undermine children’s linguistic knowledge (Hamburger and Crain, 1982; Crain and Thornton, 1998; Gualmini, 2004, 2005; Gualmini et al., 2008). It is generally acknowledged that it is pragmatically appropriate to ask a manner *zenme* question when a potential tool or method (aka the comitativity of the action) is explicitly detectable in the context; and it is felicitous to raise a causal *zenme* question when an on-going event contradicts the speaker’s expectation (aka the counter-expectation prerequisite), thereby leading the speaker to ask what has led to the unexpected event (Tsai, 2008). So, to satisfy the felicity conditions of the two uses of *zenme*, the comitativity of the action and the counter-expectation prerequisite were clearly established in the stories. In the example story, to satisfy the felicity condition on the use of the causal question ‘why did the fish hide the treasure box for the frog?,’ a counter-expectation was established when the fish finally agreed to help the frog despite the fact that he was often bullied by the frog. Meanwhile, the fish indicated that his shell was an ideal tool to perform the hiding action, so as to satisfy the felicity condition on the use of the manner question ‘how did the fish hide the treasure box for the frog?’ The second design feature is that we made available both the causal reading and the manner reading in each story. Third, to control for potential salience effects on participants’ interpretations due to the order of mention, the sequence of events that corresponded to the two readings was counterbalanced across the four stories. In two of the stories, the causal reading was established first and in the other two, the manner reading was realized first. In the example story, the causal reading was established before the manner reading.

The test sentence that was constructed for this example story was the one in (9). The filler sentence corresponding to this story was given in (11), which is a simple *wh*-question containing *shenme* ‘what.’


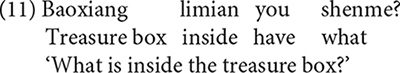


### Procedure

In this Question–Answer task, before the test session, the children were given one warm-up session, in which a puppet who appeared on a laptop computer screen asked simple questions about story settings (see Appendix B). This warm-up session was used to familiarize the participants with the task. Only those children who correctly answered all the questions in this session were included in the test session. In addition, children who were too shy to interact with the puppet were not invited to the test session. In Experiment 1, all the child participants correctly answered the warm-up questions and then proceeded to the test session. In the test session, the experimenter acted out stories in front of the child participant using toy props, and the puppet watched the stories alongside the child participant. In the story, the puppet would ask the child a question using a test sentence, and the child was instructed to answer the question. The test sentences were prerecorded and were presented to the participant through the laptop computer connected to an external speaker to make it appear that the puppet was talking. It was made clear to the participants that the puppet did not always pay close attention to the story and thus sometimes he might get confused about what happened in the story. When he was unsure about what happened in the story, he would ask a question. On each trial, the participants’ task was to help the puppet better understand the story by answering the questions for the puppet.

The participants were introduced to the task individually and then tested individually in a quiet room in the Kindergarten. The adult participants were tested using a videotaped version of the same task. They heard the same stories as presented to the child participants. At the end of each story, they were also instructed to answer the questions.

### Predictions

If participants interpreted *zenme* questions as manner questions, then in response to the puppet’s question in (9), they would be expected to provide an answer “He hid the treasure box into his shell.” If, on the other hand, participants interpreted *zenme* questions as causal questions, then they should answer “Because the frog gave him a gold coin” in response to the same question.

### Data Treatment

We coded the responses as manner reading when the participant answered the question by indicating a manner (e.g., *Xiaoyu ba baoxiang cang zai beike li* ‘The fish hid the treasure box into the shell,’ or *Ta dakai beike fang jinqu* ‘He opened the shell and put it in’), and those as causal reading when participants answered the question by indicating a cause (e.g., *Yinwei qingwa gei ta yige jinbi*, ‘Because the frog gave him a gold coin’ or *Yinwei xiaoyu hen xihuan jinbi* ‘Because the fish liked the gold coin very much’). Other irrelevant responses or failure to provide any responses were coded as “others.”

We wish to note that we did not ask children to limit their answers to a certain number of sentences or to a certain length, yet they responded to both the manner and causal questions mainly using a simple sentence as indicated in the example responses. Children’s responses of both types are generally equal in length and in structural complexity. Thus, it seems quite unlikely that children’s preference for the manner reading was due to that manner responses were often associated with longer answers and thus were easier to be produced by children^2^.

### Results and Discussion

All the participants responded correctly to the filler questions 100% of the time. So, their data were all included in the final analyses.

**FIGURE 3:**
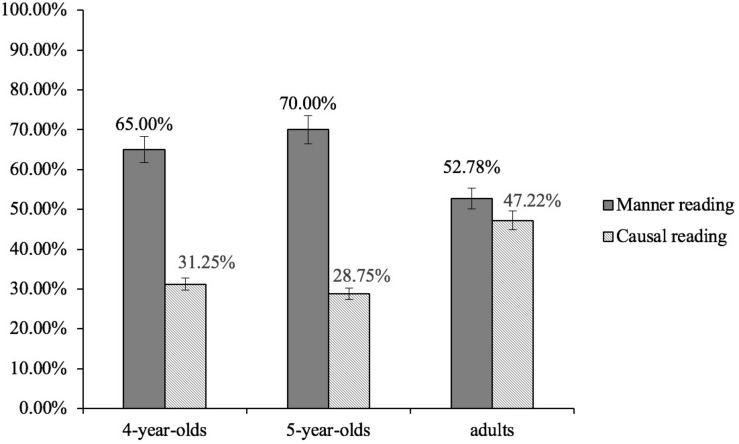
Mean proportions of the two readings by the three age groups. Error bars indicate SEs.

For each participant, we first calculated the number of responses indicating a manner reading and the number of responses corresponding to a causal reading. We then computed the proportions of the two types of responses (i.e., the two readings). Figure 3 shows the mean proportions of the two readings by the three age groups^3^. As indicated in Figure 3, all the three groups assigned two readings to *zenme* questions. The 4-year-olds assigned a manner reading to *zenme questions* 65.00% of the time, and the 5-year-olds and the adults did so 70.00% and 52.78% of the time, respectively. The 4-year-olds assigned a causal reading to *zenme* questions 31.25% of the time, and the 5-year-olds and the adults did so 28.75% and 47.22% of the time, respectively. The 4-year-olds and the 5-year-olds assigned more manner readings than causal readings to *zenme questions*, whereas the adults assigned the two readings equally often.

To assess the response patterns among the three groups statistically, generalized linear mixed models (GLMMs) were applied. We conducted the fitting process via functions *lmer* from package *lme4* (v1.1-12) (Bates et al., 2015b) of the R (v3.2.5) software environment (R Development Core Team, 2017). Satterthwaite approximation implemented in the package *lmerTest* (Kuznetsova et al., 2017) was used to estimate the degrees of freedom. The significance of predictors was determined using an alpha-level of 0.05.

We treated the manner and the causal responses as two levels of the dependent variable when computing statistical models. In the full model, the fixed effects included the participants’ group; the random effects included both items and participants, where both their intercepts and slopes were allowed to vary among all the fixed effects (Baayen et al., 2008; Barr et al., 2013). The full model’s complexity was then reduced to see whether the reduced model could explain the same variance as the full model (Bates et al., 2015a). If it could, we would accept the simplified model.

The model results showed that for the two child groups, the proportion of the manner reading was significantly higher than that of the causal reading (the 4-year-olds: β = 1.10, *SE* = 0.27, *z* = 4.56, *p* < 0.001; the 5-year-olds: β = 1.22, *SE* = 0.33, *z* = 5.52, *p* < 0.001). By contrast, for the adults, there was no significant difference between the proportion of the manner reading and that of the causal reading (β = 0.13, *SE* = 0.23, *z* = 0.51, *p* > 0.05).

The model results also revealed that age group (the adult group was treated as the baseline) was a reliable predictor for the participants’ responses of both types. There was a significant difference in the proportion of manner readings between the 4-year-olds and the adults (β = 1.05, *SE* = 0.22, *z* = 2.19, *p* < 0.05), and between the 5-year-olds and the adults (β = 1.07, *SE* = 0.20, *z* = 2.24, *p* < 0.05). In addition, there was a significant difference between the 4-year-olds and the adults (β = 1.07, *SE* = 0.18, *z* = 2.61, *p* < 0.01) and between the 5-year-olds and the adults (β = 1.13, *SE* = 0.16, *z* = 2.72, *p* < 0.01).

The response patterns of three age groups, as shown in Figure 3, were supported by the statistical modeling. The findings are evidence that Mandarin-speaking children as young as 4 years of age access both manner and causal readings of *zenme*^4^. The ambiguity of *zenme* as discussed in the theoretical literature has been experimentally supported by data from both child and adult Mandarin.

However, it is also worth pointing out that the 4-year-olds and the 5-year-olds assigned the manner reading to *zenme* significantly more often than they did with the causal reading, but the adults assigned the two readings equally often. The findings suggest that unlike adults, Mandarin-speaking children prefer the manner reading over the causal reading even though both readings are available to them.

## Experiment 2

Experiment 2 was designed to investigate whether young Mandarin-speaking children are able to use the syntactic positions of *zenme* relative to modal verbs to disambiguate the two readings of *zenme*, and whether children exhibit a developmental trajectory when acquiring the mapping relations between the syntactic positions of *zenme* and its corresponding semantic interpretations.

As discussed, the syntactic positions of *zenme* as relative to modal verbs can be used to disambiguate between the causal reading and the manner reading. For example, when *zenme* is structurally lower than the modal verb *hui* ‘will/would,’ as in (12a), it yields a manner reading; but when *zenme* is structurally higher than the modal verb *hui*, as in (13a), it gives rise to a causal reading. Experiment 2 used minimal pairs as in (12a) and (13a).


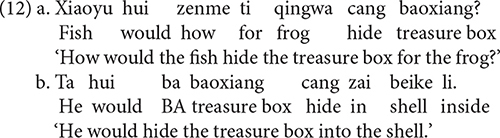



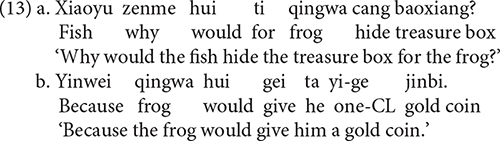


### Participants

One hundred and thirty monolingual Mandarin-speaking children participated in Experiment 2^5^. They were divided into three age groups. Forty children were between the age of 3;4 and 3;11 (22 boys and 18 girls, mean age = 3;9), 40 children were between 4;1 and 4;11 (23 boys and 17 girls, mean age = 4;7), and 50 children were between 5;0 and 5;11 (33 boys and 17 girls, mean age = 5;6). The child participants were recruited from Beijing Taolifangyuan Kindergarten and had no reported history of speech, hearing or language disorders. In addition, 40 Mandarin-speaking adults (age range 19–31, mean age = 23) were tested as controls. They were students at Tsinghua University, and had no self-reported speech or hearing disorders. None of the participants had taken part in Experiment 1.

### Procedure

We used a Question–Answer task. The experimental procedure in this experiment was identical to Experiment 1.

### Materials and Design

The four stories used in Experiment 2 were identical to those used in Experiment 1. But Experiment 2 used different test structures. For each story, two types of test sentences were created, one containing *zenme* in a structurally lower position than the modal verb *hui* [see (12a)], and one with *zenme* in a structurally higher position than *hui* [see (13a)]. In addition to the two test sentences, one filler sentence was created for each story. The filler sentences were the same simple *wh*-questions containing *shenme* ‘what’ as in Experiment 1. See Appendix C for all the test and filler sentences.

We used a between-participants design. Half of the participants in each of the four age groups (i.e., twenty 3-year-olds, twenty 4-year-olds, twenty five 5-year-olds and twenty adults) heard the sentence in (12a) and the other half heard the sentence in (13a). Across the trials, one group of participants heard four test sentences containing *zenme* in a structurally lower position than *hui* (Group A), and the other group were presented with four test sentences containing *zenme* in a structurally higher position than *hui* (Group B). In addition, both groups heard four filler sentences containing *shenme* ‘what,’ as in (11).

### Predictions

If children can use the syntactic positions of *zenme* relative to modal verbs to distinguish the causal reading from the manner reading, then they should interpret sentences containing *zenme* in a structurally lower position than *hui* as manner questions, and they should interpret sentences containing *zenme* in a structurally higher position than *hui* as causal questions. On the example trial, in response to (12a), children would be expected to provide an answer in (12b) indicating the manner in which the fish would hide the treasure box (i.e., he would hide the treasure box into the shell); and in response to (13a), children should provide an answer in (13b) indicating the reason why the fish would hide the treasure box for the frog (i.e., because the frog would give him a gold coin).

### Results and Discussion

**FIGURE 4:**
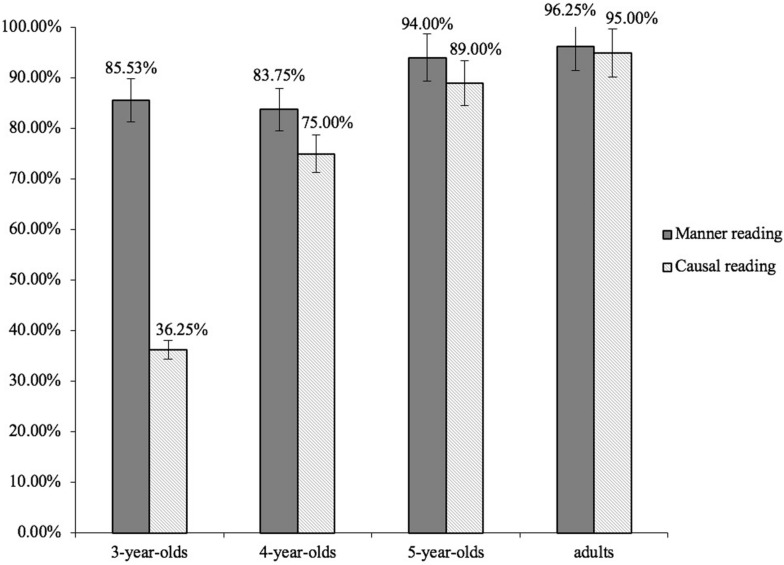
Mean proportions of the two readings in response to the two structures by the four age groups. Solid bars indicate the proportions of manner readings in response to manner questions. Lined bars indicate the proportions of causal readings in response to causal questions. Error bars indicate SEs.

We excluded data of one 3-year-old child in Group A from the final analyses, because he failed on two of the four filler questions. The rest of the participants responded correctly to at least three of the four filler questions, and thus were included in the analyses. For each participant we first coded their responses using the same criteria as in Experiment 1. We calculated the number of responses indicating a manner reading and the number of responses indicating a causal reading in response to the two structures. We then computed the proportion of the two types of readings in each condition. Figure 4 summarized the results. As indicated in the solid bars in Figure 4, when responding to sentences containing *zenme* in a structurally lower position than *hui*, as in (12a), all the age groups consistently provided correct manner interpretations. The adults provided answers indicating a manner interpretation 96.25% of the time, the 3-year-olds, 4-year-olds, and the 5-year-old did so 85.53%, 83.75%, and 94.00% of the time, respectively. When responding to sentences containing *zenme* in a structurally higher position than *hui*, as in (13a), different response patterns were observed among the four age groups. The adults and the 5-year-olds consistently provided correct causal interpretations 95.00% and 89.00% of the time, respectively. The 4-year-olds provided causal interpretations 75.00% of the time. But the 3-year-olds provided causal interpretations only 36.25% of the time, and 49.00% of the time they provided answers indicating a manner interpretation.

Again, generalized linear mixed models were applied to assess the response patterns among the four age groups. We used the same fitting process as in Experiment 1. The best-fitting model treated age group (i.e., four age groups) and sentence type (two types of structures) as fixed effects, with random intercepts and slopes for both participants and items.

The model results revealed the 5-year-olds provided more causal interpretations than the 4-year-olds (β = 1.07, *SE* = 0.21, *z* = 2.51, *p* < 0.01) and the 3-year-olds (β = 1.09, *SE* = 0.24, *z* = 4.49, *p* < 0.001); and the 4-year-olds provided more causal interpretations than the 3-year-olds (β = 1.11, *SE* = 0.19, *z* = 2.50, *p* < 0.01).

The model results also showed that in response to sentences containing *zenme* in a structurally lower position than *hui* (manner questions), no significant effect of age group (the adult group was treated as the baseline) was observed among the four age groups in the proportion of their manner readings (3-year-olds versus adults: β = 0.09, *SE* = 0.13, *z* = 1.03, *p* > 0.05; 4-year-olds versus adults: β = 0.08, *SE* = 0.17, *z* = 0.67, *p* > 0.05; 5-year-olds versus adults: (β = 0.08, *SE* = 0.19, *z* = 0.59, *p* > 0.05). By contrast, in response to sentences containing *zenme* in a structurally higher position than *hui* (causal questions), age group was a reliable predictor of their responses. More causal interpretations were observed in the adults than in the 4-year-olds (β = 1.16, *SE* = 0.25, *z* = 2.77, *p* < 0.01) and the 3-year-olds (β = 1.19, *SE* = 0.24, *z* = 4.87, *p* < 0.001), but no significant difference was found between the adults and the 5-year-olds (β = 0.11, SE = 0.17, *z* = 0.70, *p* > 0.05).

The findings suggest that the 5-year-old Mandarin-speaking children, like the adults, are able to use the structural relations between *zenme* and modal verbs to disambiguate the two readings of *zenme*. The 3-year-olds, however, are still in the stage of working out how the syntactic positions of *zenme* are mapped onto the relevant semantic interpretations. When presented with structures where *zenme* is structurally higher than *hui*, the 3-year-olds tend to interpret them in the same way as structures where *zenme* is structurally lower than *hui*. The 4-year-olds are in a transitional stage and they are generally able to map the syntactic positions onto the corresponding semantic interpretations of *zenme*, although they are not quite as good as the 5-year-olds. In general, children exhibit a developmental trajectory when acquiring the mapping relations between the syntactic positions of *zenme* and its corresponding semantic interpretations. The critical change occurs at around 4 years of age.

## Corpus Analysis

To investigate whether parental input had an impact on children’s preference for the manner reading over the causal reading, we analyzed the parental input in the CHILDES Database (MacWhinney, 2000). More specifically, we were interested to see if the manner reading is a more frequent use of *zenme* than the causal reading by caretakers, i.e., whether there is sufficient parental input for the manner reading of *zenme* but not for the causal reading of *zenme*.

We did a corpus survey of 62,309 adult utterances of 10 children in the Beijing Corpus^6^ in the CHILDES Database (Tardif, 1993, 1996). Table 1 reports the token frequencies of the causal *zenme* and the manner *zenme* in bare forms and in constructions containing the modal verb *hui* for each child. As illustrated in Table 1, both the tokens of the manner *zenme* and the causal *zenme* in bare forms are limited in number. For these ten children, there was no significant difference in the adult input between the token frequencies of the manner *zenme* and the causal *zenme* in bare forms (*z* = 0.26, *p* > 0.05). In addition, we found that the manner *zenme* constructions (with *zenme* in a structurally lower position than the modal verb *hui*) and the causal *zenme* constructions (with *zenme* in a structurally higher position than the modal verb *hui*) are even scarcer, with only five instances of causal *zenme* constructions (e.g., *Zenme hui teng a?* ‘Why would it hurt?’), and not a single instance of manner *zenme* constructions. Again, for these ten children, no significant difference was observed between the token frequencies of the manner *zenme* constructions and the causal *zenme* constructions (*z* = 1.89, *p* > 0.05).

**TABLE 1:** Token frequencies of causal *zenme* and manner *zenme* in bare forms and in constructions containing the modal verb *hui* in Beijing Corpus (Tardif, 1993, 1996).

Children	Causal *zenme*	Manner *zenme*	*zenme* > *hui*	*hui* > *zenme*
BB	53	54	2	0
CX	26	13	1	0
HY	17	25	0	0
LC	27	18	0	0
LL	39	26	1	0
LXB	48	34	1	0
TT	21	23	0	0
WW	26	29	0	0
WX	48	68	0	0
YY	51	54	0	0
**Total**	**356**	**344**	**5**	**0**

*The bold values indicate the total numbers of token frequencies of causal zenme and manner zenme in bare forms and in constructions containing the modal verb hui in Beijing Corpus, respectively.*

## General Discussion

The present paper sought to investigate children’s acquisition of *zenme* in Mandarin. Two experiments were conducted. Experiment 1 was designed to see whether young Mandarin-speaking children understand that the *wh-*adjunct *zenme* is ambiguous between a causal reading and a manner reading. Experiment 2 examined whether young children are able to use the syntactic positions of *zenme* relative to modal verbs to disambiguate the two readings of *zenme* and whether children exhibit a developmental trajectory when acquiring the mapping relations between the syntactic positions of *zenme* and its corresponding semantic interpretations.

The findings of Experiment 1 show that Mandarin-speaking children as young as 4 years of age access both the manner and the causal readings of *zenme*, but they prefer the manner reading over the causal reading. The findings of Experiment 2 demonstrated that children exhibit a developmental trajectory when acquiring the mapping relations between the syntactic positions of *zenme* and its corresponding semantic interpretations. 5-year-old children are able to use the structural relations between *zenme* and modal verbs to disambiguate the two readings of *zenme*; 3-year-olds, however, are still in the stage of working out how the syntactic positions of *zenme* are mapped onto the relevant semantic interpretations. When presented with structures where *zenme* is structurally higher than *hui*, 3-year-olds tend to interpret them in the same way as structures where *zenme* is structurally lower than *hui*. The findings suggest that young Mandarin-speaking children have a preference for the manner reading over the causal reading. The manner reading seems to be the default reading of *zenme*.

At this point, an interesting question to ask would be why the manner reading is the default reading for young children. This is also the point to discuss how the data can inform us about the debate between the two competing approaches of child language acquisition, i.e., which approach can better explain the experimental findings.

On the usage-based account, the more frequent use of *zenme* in the linguistic input should be acquired earlier than the less frequent one. In other words, children acquire the manner use of *zenme* earlier than the causal use of *zenme*, because they are exposed to more manner uses than causal uses of *zenme* in the input. However, the findings of the corpus analysis showed that there was no significant difference between the token frequencies of the manner *zenme* constructions and the causal *zenme* constructions. The discrepancy between the adult input and the children’s interpretation of *zenme* in the two experiments provides evidence that input alone cannot explain Mandarin-speaking children’s acquisition of the two readings of *zenme*.

In contrast, on the UG-based approach, children acquire the manner *zenme* question earlier than the causal *zenme* question, because the causal *zenme* question involves the CP layer and the manner *zenme* question relates to the VP layer. The CP layer at the left periphery of the structure is often associated with discourse functions, and the mapping between syntax/semantics and discourse/pragmatics is not well established until at a relatively late stage of language acquisition (Rizzi, 1994; Müller et al., 1996; Radford, 1996; Grinstead, 1998; Platzack, 2001; Marinis, 2004; Spinner and Grinstead, 2006). On this account, children’s preference for the manner reading over the causal reading can be easily explained. Children’s initial tendency to analyze *zenme* as manner *zenme* rather than causal *zenme*, is presumably due to their ability to represent the VP layer (corresponding to the manner *zenme*) but their non-adult representation of the CP layer (corresponding to the causal *zenme*). Overall, two features of the UG-based approach make it a more suitable account for young Mandarin-speaking children’s acquisition of the two readings of *zenme.* One is the acknowledgment of the discrepancy between the linguistic knowledge acquired by young children and the linguistic input they have been exposed to, and the second is the emphasis of the importance of hierarchical structure in children’s acquisition of linguistic knowledge.

Before concluding, we wish to point out that in addition to structural reasons, the manner reading default might also have to with some lexical factors. In Mandarin, there is another *wh*-word *weishenme* ‘why,’ which is often used to question the cause of an event. It is possible that young children initially use *weishenme* ‘why’ to inquire about reasons and *zenme* ‘how’ to inquire about manners. There might be a division of labor between *zenme* and *weishenme* in the early lexicon.

We also wish to point out that our corpus analysis was based on the parental input in the Beijing Corpus in the CHILDES Database, which might be limited in scope and content. Although it is the standard practice to analyze the parental input in the CHILDES Database when discussing the role of input in child language acquisition (Crain et al., 2017; Yang et al., 2017; Koring et al., 2020), we acknowledge that to gain a comprehensive understanding of the role of input, further research is required to investigate children’s other sources of input including their story books, textbooks, cartoons and interactions with teachers at kindergartens and at early childhood education centers.

To summarize, our experimental findings provide empirical evidence for the ambiguity of the *wh-*adjunct *zenme* discussed in the theoretical linguistics literature. Young children as adults access both the manner and the causal readings of *zenme*, although they exhibit a strong preference for the manner reading. The present paper provides a good example of convergence of child language acquisition and theories of linguistic structures. Although children initially assign a manner reading to *zenme*, by age 4 or 5 they become able to use syntactic cues, i.e., the syntactic positions of *zenme* relative to modal verbs, to arrive at the correct interpretation of *zenme*. In addition, the findings, to some extent, can inform us about the debate of the two competing approaches to child language acquisition: the UG-based approach and the usage-based approach. In the current study, the patterns exhibited by young children in their acquisition of *zenme* questions can be better explained by the UG-based approach than the usage-based approach.

## Data Availability Statement

The data that support the findings of this study are available from the corresponding author upon request.

## Ethics Statement

The study was approved by the Ethics Committee of the School of Medicine, Tsinghua University, 20170018. Written informed consent has been obtained from each child participant’s parents and each adult participant.

## Author Contributions

JL and PZ contributed equally to this work.

## Conflict of Interest

The authors declare that the research was conducted in the absence of any commercial or financial relationships that could be construed as a potential conflict of interest.

## Footnotes

1 Tsai (2008) and Tang (2011) argued that when *zenme* precedes the subject NP such as in *Zenme ni qu Beijing?* ‘How come you went to Beijing?’ it expresses a strong exclamatory construal. When *zenme* is placed in this position (i.e., at ForceP), its illocutionary force changes from interrogation to exclamation or denial. But the denial *zenme* is not our concern in the present study. We only focus on the contrast between causal *zenme* and manner *zenme*.

2 This section responds to a concern raised by one of the reviewers about whether children’s responses indicating a manner reading were longer than those indicating a causal reading, showing that children’s preference for the manner reading was presumably due to that manner responses were often associated with longer answers and thus were easier to be produced by children. We appreciate the opportunity to clarify the point, and have now made clear that children’s responses of both types are generally equal in length and in structural complexity.

3 One 5-year-old child and one 4-year-old child provided an uninterpretable answer in response to one of the four test sentences, respectively; one 4-year-old child provided irrelevant answers in response to two of the four test sentences. Thus, these four responses were not included in the final analyses.

4 One reviewer raised the concern that our results did not necessarily indicate that each child understands that *zenme* is ambiguous between a manner and a causal reading, because they each gave either a manner or a causal response to *zenme*. However, we wish to note that even the adults answered the *zenme* questions by choosing one reading over the other, but post-test interviews showed that they judged the *zenme* questions to be ambiguous 100% of the time. We could not ask children explicitly whether the *zenme* sentences were ambiguous, due to their immature metalinguistic ability, but the results clearly show that 4-year-olds as a group access both the manner and the causal reading of *zenme*.

5 In Experiment 2, two additional 3-year-olds who were unwilling to interact with the puppet in the warm-up session were not invited to the test session and thus were not included in the final sample.

6 The Beijing Corpus contains files of conversations between adults and individual child participants between the ages of 1;9.3 and 2;2.7. The computerized language analysis program (CLAN) was used to determine the number of utterances and the number of target words, as well as to identify the immediate context of the target words.

## Appendix A

Test and filler sentences in Experiment 1: (1) to (4) were test sentences, and (5) to (8) were filler sentences.

## Appendix B

Warm-up questions in both Experiment 1 and Experiment 2.

## Appendix C

Test sentences in Experiment 2: (1) to (4) were presented to Group A, and (5) to (8) were presented to Group B. The same set of filler sentences as in Experiment 1 was presented to both groups.
